# Influence of Amino Acids and Exercise on Muscle Protein Turnover, Particularly in Cancer Cachexia

**DOI:** 10.3390/cancers16101921

**Published:** 2024-05-18

**Authors:** Rashmita Pradhan, Walburga Dieterich, Anirudh Natarajan, Raphaela Schwappacher, Dejan Reljic, Hans J. Herrmann, Markus F. Neurath, Yurdagül Zopf

**Affiliations:** 1 Department of Medicine, Friedrich-Alexander University Erlangen-Nürnberg, 91054 Erlangen, Germany; rashmita.pradhan@uk-erlangen.de (R.P.); walburga.dieterich@uk-erlangen.de (W.D.); anirudh.natarajan@uk-erlangen.de (A.N.); raphaela.schwappacher@uk-erlangen.de (R.S.); dejan.reljic@uk-erlangen.de (D.R.); hans.herrmann@uk-erlangen.de (H.J.H.); markus.neurath@uk-erlangen.de (M.F.N.); 2 Hector-Center for Nutrition, Exercise and Sports, Department of Medicine 1, Friedrich-Alexander University Erlangen-Nürnberg, 91054 Erlangen, Germany

**Keywords:** cancer cachexia, inflammation, oxidative stress, amino acids, exercise

## Abstract

**Simple Summary:**

Cancer cachexia (CC) is a syndrome affecting advanced cancer patients, causing inflammation, metabolic dysfunction, and a decline in quality of life. It increases the risk of mortality. Nutritional therapies are being tested for improving muscle metabolism in cancer patients, but no special therapies have been validated in clinical practice. Studies suggest increasing muscle protein synthesis through amino acids or protein intake, and physical activity can reduce muscle atrophy. This manuscript provides an overview of the preclinical and clinical approaches for the use of amino acids with and without exercise therapy to improve muscle metabolism in cachexia.

**Abstract:**

Cancer cachexia is a multifaceted syndrome that impacts individuals with advanced cancer. It causes numerous pathological changes in cancer patients, such as inflammation and metabolic dysfunction, which further diminish their quality of life. Unfortunately, cancer cachexia also increases the risk of mortality in affected individuals, making it an important area of focus for cancer research and treatment. Several potential nutritional therapies are being tested in preclinical and clinical models for their efficacy in improving muscle metabolism in cancer patients. Despite promising results, no special nutritional therapies have yet been validated in clinical practice. Multiple studies provide evidence of the benefits of increasing muscle protein synthesis through an increased intake of amino acids or protein. There is also increasing evidence that exercise can reduce muscle atrophy by modulating protein synthesis. Therefore, the combination of protein intake and exercise may be more effective in improving cancer cachexia. This review provides an overview of the preclinical and clinical approaches for the use of amino acids with and without exercise therapy to improve muscle metabolism in cachexia.

## 1. Introduction

Cancer cachexia (CC) is a complex multifactorial syndrome marked by a loss of skeletal muscle mass, with or without body fat, occurring in around 50–80% of cancer patients and leading to death in about 20% of them [1]. CC has been categorized, based on its severity, into three progressive stages: pre-cachexia, cachexia, and refractory cachexia [2]. In pre-cachexia, less body weight loss (BWL) occurs due to metabolic changes like impaired glucose tolerance; however, in cachexia, >5% BWL occurs in six months or >2% BWL occurs with a body mass index of <20 kg/m^2^, and in refractory cachexia (BWL ≥ 20%), mostly patients are in a highly catabolic state with a life expectancy of fewer than 3 months [3]. Early screening for malnutrition, followed by proper nutritional therapy and supplementation such as proteins and amino acids, can limit complications and improve the patient’s prognosis [4].

Besides muscle and fat wasting, CC is typically associated with symptoms like anorexia, anemia, asthenia, fatigue, and insulin resistance. Muscle atrophy in cachexia leads to poor muscle strength and reduced mobility and ultimately affects the quality of the patient’s life. Studies have shown that patients with CC are more likely to experience systemic inflammation and oxidative stress [5,6]. To counteract increased catabolic process in patients with CC, an improvement in muscle metabolism through exercise/sports in combination with an adapted protein intake is required [7]. From a metabolic perspective, patients with cachexia develop a wide range of metabolic dysfunctions, including the production of reactive oxygen species (ROS), autophagy deregulation, and mitochondrial dysfunction [6]. In addition, cachexia is associated with both the activation of immune cells and endocrine signaling pathways. These changes can negatively impact tumor growth and skeletal muscle metabolism. The alteration can be caused by several factors, including increased levels of cytokines, adrenaline, insulin, and insulin-like growth factor 1 (IGF-1) resistance. As a result, energy expenditure and heat generation may also be increased [8]. Insulin and IGF-1 induce muscle growth by stimulating the phosphoinositide 3 kinase (PI3K)/Akt/mammalian target of rapamycin (mTOR) pathway, where Akt activation induces mTOR (mammalian target of rapamycin) signaling, an essential pathway for healthy muscle cell growth [1]. The inflammatory mediators released in cancer lead to an imbalance between muscle protein synthesis and breakdown. Cancer-specific therapies such as chemotherapy, radiotherapy, and antibody therapies can further exacerbate this imbalance by disrupting protein synthesis and promoting muscle breakdown. To stabilize the muscles and body composition, international oncological guidelines recommend a high-protein diet of 1.2–2.0 g/kg body weight for cancer patients [9,10]. A high intake of amino acids also appears to be a promising strategy for the treatment of CC [11]. In addition to a high-protein diet and/or the use of amino acids, exercise training appears to have decisive positive effects on muscle metabolism, physical performance, and the patient’s quality of life. The safety and feasibility of exercise have also been demonstrated in patients with advanced cancer [12,13]. Nutritional therapy alone, with or without additional pharmacological measures, does not appear to be sufficient for the treatment of anorexia or muscle wasting in some cases [13]. There is increasing evidence that the combination of diet and exercise is the most effective treatment for muscle wasting in cancer patients [14,15,16]. However, more targeted studies are needed to determine the optimal training dose and the amount of protein or amino acid intake required, depending on the different types of cancer.

In this review, we will outline the known molecular pathogenesis of CC and possible therapeutic approaches for targeted nutrition and exercise therapy. We will explain the importance of selected amino acids and their influence on muscle protein synthesis in healthy individuals and patients with CC. We will also discuss the potential of combining exercise and nutritional therapy/amino acid application as an intervention approach in experimental animal models of cachexia and clinical trials. This review stands out from the existing literature by providing an up-to-date comprehensive overview of the molecular mechanisms behind CC and suggesting precise therapeutic approaches. These approaches involve recent data on combining exercise with supplementation of the most-studied amino acids, recognizing their synergistic potential in mitigating muscle wasting in CC.

## 2. Metabolic Dysfunction in Cancer Cachexia

CC is a multiorgan syndrome in which pathogenesis is affected by several factors. Despite the limited understanding of the molecular mechanisms associated with the development and progression of CC, it has been demonstrated that the interaction between cancer cells and other organs, particularly skeletal muscle and fat mass, significantly impacts the body composition of cancer patients [14]. Recent investigations showed that besides skeletal muscle and fat tissues, the brain, liver, gut, and heart are also directly involved in the cachectic process, leading to muscle wasting [1]. In this section, we will provide a brief overview of the molecular alterations that occur during the development of CC.

### 2.1. Muscle Wasting in Cancer Cachexia

The imbalance between the anabolic and catabolic metabolism due to inflammation, physical inactivity, and inadequate nutrition ultimately results in muscle wasting, which is a prominent feature of CC. The activation of several intracellular signaling pathways by extracellular ligands are involved in muscle protein turnover and wasting process, e.g., pro-inflammatory and pro-cachectic cytokines like tumor necrosis factor-α (TNF-α), interleukin-1 (IL-1), and IL-6 have been shown to activate the nuclear factor kappa B (NF-κB) pathway as well as the Janus kinase signal transducer (JAK-STAT) pathways [15]. The pro-inflammatory cytokines upregulate the muscle-specific ubiquitin-mediated proteasome degradation pathway (UPP), especially the expression of E3 ubiquitin ligases, including muscle ring-finger protein 1 (MuRF1) and muscle atrophy F-box protein (MAFbx, Atrogin 1, or FBXO32). These E3 ubiquitin ligases facilitate muscle protein breakdown and inhibit protein synthesis [16,17]. Besides cytokines, transforming growth factor-β (TGF-β) superfamily members like myostatin and activin are also involved in the muscle atrophying process. Myostatin increases protein degradation and reduces protein synthesis in skeletal muscle by activating the SMAD complex, which is formed from Caenorhabditis elegans and mothers against decapentaplegic, and the mitogen-activated protein kinase (MAPK) pathway [18]. In addition to muscle protein degradation, protein synthesis pathways are also altered in patients with CC [19,20]. The primary pathway for the synthesis of proteins is known as the PI3K/AKT/mTOR signaling pathway, which is activated by insulin and IGF1. The activation of these pathways triggers the phosphorylation of downstream targets, including p70S6 kinase (p70S6K) and PHAS-1/4E-BP1 [21]. In addition, AKT phosphorylation inhibits the FoxO family of transcription factors (FoxOs: FoxO1, FoxO3, and FoxO4), which is responsible for the upregulation of E3 ligases, MuRF1, and Atrogin-1 and the corresponding degradation of muscle protein [22].

At the cellular level, muscle protein degradation is increased through the induction of apoptosis [23], calcium-activated protease calpains [24,25], lysosomal proteolysis and overactivation of autophagy [26], mitochondrial dysfunction [27], and the direct effect of cancer chemotherapy [28]. Increased molecular gene expression of autophagic genes, such as BNIP3 and LC3B-II, is observed in cancer muscle biopsies. This is associated with increased proinflammatory cytokines and activation of NF-κB in the muscles [29]. Recently, in preclinical studies, several novel molecules have been found to trigger muscle wasting in cancer, e.g., TNF-related weak inducer of apoptosis (TWEAK) [30], TNFα receptor adapter protein (TRAF6) [31], leukemia inhibitory factor [32], high mobility group box 1 (HMGB1) [33], and tumor-derived microribonucleic acid (miRNAs) [34,35].

### 2.2. Oxidative Stress in Cancer Cachexia

In eukaryotic muscle cells, several organelles like mitochondria, sarcoplasmic reticulum, and sarcolemma (the cell membrane of myocytes) are involved in producing ROS, which are essential for the initial response to pathogens. Moreover, enzymes such as nicotinamide adenine dinucleotide phosphate (NADPH) oxidase and xanthine oxidase (XO) also contribute to the production of ROS. Additionally, cancer increases the levels of various pro-inflammatory cytokines, including IL-1, IL-6, and TNF-α, which contribute to the development of cachexia and oxidative damage. In particular, TNF-α stimulates ROS production and activates NADPH oxidase in mitochondria. When the production of oxygen and nitrogen species (ROS/RNS) surpasses the cellular antioxidant system, this leads to oxidative stress. Studies have demonstrated that oxidative stress could impair the functionality of cellular structures and proteins by causing damage to lipids, DNA, and proteins themselves [6].

Excessive accumulation of mitochondrial uncoupling proteins (UCPs) such as UCP2 and UCP3 can impair the mitochondrial membrane potential in patients with CC, thus leading to increased production of mitochondrial ROS. Oxidative stress is identified as one of the factors that exacerbate mitochondria damage in such patients [36,37]. The activation of ROS/RNS triggers several intracellular signaling pathways, including the redox-sensitive transcription factors NF-κB and activator protein 1 (AP-1) [6]. NF-κB participates in impaired myogenesis and activates intracellular proteolysis [38]. Additionally, elevated levels of ROS/RNS can oxidize specific cysteine residues in proteins, ultimately causing protein degradation [39].

### 2.3. Altered Energy Balance in Cancer Cachexia

In CC patients, resting energy expenditure (REE) increases due to the cancer and the cancer-specific therapy. The simultaneous reduced energy intake and/or insufficient protein intake lead to wasting syndrome [40]. Several mechanisms are responsible for the increased energy expenditure and, thereby, the loss of body weight. For example, the recycling of lactic acid between liver and cancer cells plays a crucial role in energy inefficiency [1]. Cancer cells metabolize glucose into lactate also in the presence of oxygen, the so-called Warburg effect. The lactate is subsequently transported to the liver and converted back by lactate dehydrogenase into pyruvate which is further converted into glucose. This results in a vicious circle with the uptake of glucose from tumor cells metabolizing into lactate and, in turn, the stimulation of glucose production in liver cells thus contributing to increased energy consumption [41].

Glucose tolerance in cancer patients decreases due to the overproduction of glucose by liver cells or decreased peripheral glucose utilization. It has been shown that intestinal dysfunction in Wistar rats results in the altered expression of intestinal glucose transporters, e.g., GLUT1, GLUT2, and GLUT5, ultimately affecting energy homeostasis [42]. Altered intestinal gluconeogenesis, which is a recently discovered regulator of the central control of glucose and energy balance, might be a contributing factor to the energy imbalance seen in cachexia [43].

Another mechanism that participates in energy inefficiency is the disruption of mitochondrial ATP synthesis through the activation of uncoupling proteins (UCPs) [1]. These proteins have been found to disrupt the proton electrochemical gradient between the outer and inner membranes of mitochondria, which can impact the production of ATP. Both murine models and patients with cachexia have shown an increase in UCP2 and UCP3 proteins in skeletal muscles and an increase in UCP1 in brown adipose tissue [1,37,44].

### 2.4. Insulin Resistance in Cancer Cachexia

Insulin resistance represents a significant metabolic disturbance observed during cachexia [45,46]. Chronic exposure to pro-inflammatory cytokines, TNF-α, IL-6, and insulin-growth-factor-binding proteins (IGFBPs) has been reported to induce insulin resistance by directly impairing insulin signaling activation while simultaneously fueling tumor activation [47,48]. This impairment of insulin action in skeletal muscle disrupts the pathways like PI3K/Akt/mTOR1 that are essential for protein synthesis, thereby leading to muscle loss [46,49]. Notably, inactivation of the mTOR1 pathway was shown to be due to impaired IGF-1-dependent signaling in C26-induced murine models [50]. Moreover, findings from a Drosophila model have reported that IGFBP and its homolog, ImpL2, interrupt systemic insulin signaling, which induces insulin resistance and skeletal muscle loss [51]. This highlights the significance of insulin resistance in cachexia, underscoring its detrimental impact on the prognosis of patients with CC.

### 2.5. Adipose Tissue Wasting and Lipid and Fat Burning in Cancer Cachexia

Along with skeletal muscle loss, damage to white adipose tissue (WAT) and an altered lipid metabolism play key roles in the pathogenesis of CC. The WAT becomes depleted through several processes in CC; e.g., the activation of hormone-sensitive lipase (HSL) increases lipolysis and thus degrades triacylglycerols to glycerol and free fatty acids [52]. In addition to this massive lipolysis, the activity of the enzyme lipoprotein lipase (LPL) decreases, which impairs the uptake of fatty acids to the WAT. There is also a reduction of lipogenesis from glucose, which ultimately participates in adipose tissue wasting [1]. Recent investigations showed that WAT cells undergo a browning process during cancer cachexia, where they are converted into brown adipose tissue (BAT)-like cells [53,54]. The conversion of WAT to brown adipose tissue (BAT) can be activated by inflammatory mediators such as IL-6, as well as tumor-driven molecules like parathyroid hormone-related protein (PTHRP). Remarkably, studies have demonstrated that the loss of adipose tissue and an increase in BAT tissue are predictive of poorer survival outcomes in cancer patients [55,56]. Furthermore, in addition to human studies, the upregulation of markers indicative of BAT, such as PR domain zinc finger protein 16 (PRDM16) and uncoupling protein 1 (UCP1), could also be elucidated in the C26-induced cachexia murine model [57].

## 3. Role of Amino Acids in Combatting Cancer Cachexia-Induced Inflammation and Mitochondrial Dysfunction

Amino acids (AAs) maintain several cellular metabolic pathways, which are crucial for development and growth. Based on their necessity, AAs are categorized into three groups—essential, conditionally essential, and non-essential amino acids. Essential amino acids (EAAs) cannot be synthesized naturally by mammals and need to be obtained through dietary sources; conditionally essential amino acids (CEAAs) are only essential during illness and stress; and non-essential amino acids (NEAAs) are generally produced within our body [58]. It has been widely studied that EAAs, particularly leucine, are strong stimulators of muscle protein synthesis in healthy individuals. The therapeutic effect of individual amino acids or amino acid mixtures on various diseases has also been investigated. This is often performed as a therapeutic strategy for conditions that involve inflammation, oxidative stress, and altered energy balances including sarcopenia and metabolic diseases [59].

Our review mainly focuses on AAs, including branched-chain amino acids (BCAAs), L-leucine, β-hydroxy methyl butyrate (HMB), glutamine, arginine, tryptophan, and the amino acid derivative carnitine in CC. These specific AAs have received significant attention for their health benefits, whereas other AAs have remained relatively understudied. The beneficial effects of these mentioned AAs during cachexia are in detail described in the subsequent paragraphs.

Several studies showed the effect of branched-chain amino acids (BCAAs) on muscle protein metabolism, among which leucine has been studied the most [60]. It has been suggested that leucine supplementation can impact protein turnover by activating the mTOR pathway, leading to upregulation of protein translation, and reducing muscle protein degradation during CC [61,62,63]. In an experimental cachexia model of Wistar rats with Walker-256 tumor implant, a leucine-rich diet (18% protein plus 3% leucine) exhibited increased muscle protein synthesis. The consumption of a diet high in leucine can reduce protein degradation in mice with tumors. In a specific mouse model with CC that was induced by inoculation of mice with C26-tumours, enrichment of the mice diet with 8 g/kg of leucine helped to preserve up to 20% of their skeletal muscle mass [64]. In cachexia-induced mice, after supplementation of leucine, isoleucine, or valine at 1 g/kg body weight, leucine and valine showed an increase in skeletal muscle mass with an increase in the protein synthesis rate and leucine also exhibited a decrease in the protein degradation [65]. In the same study, where C2C12 myotubes were exposed to proteolysis-inducing factor (PIF), leucine supplementation also reduced PIF-related protein degradation [65]. Along with protein degradation, impaired mitochondria function is one of the major factors during skeletal muscle atrophy [66]. Cancer cachexia can reduce the expression of PGC-1α and genes involved in the ROS defense system, such as superoxide dismutase 1 and 2 and glutathione peroxidase (GPx1), consequently leading to oxidative damage. However, BCAAs supplementation has been shown in the mitigation of oxidative damage by increasing nitric oxide (NO) production through endothelial NO synthase (eNOS), and inducing expression of PGC-1α, superoxide dismutase (SOD), and GPx1, thereby promoting cellular survival [67,68]. In comparison however, it has been reported that in isolated cultured peripheral blood mononuclear cells (PBMCs), a very high concentration of BCAAs (10 mmol/L) promotes oxidative stress through increased ROS production and induces inflammation by activating the NF-κB pathway [69]. Another study has shown that increased BCAA levels can induce inflammation and oxidative stress in endothelial cells, which can in consequence affect inflammatory cell adhesion and endothelial dysfunction [69]. Therefore, more studies are needed to determine the appropriate concentrations of these amino acids for muscle protein synthesis in patients with cancer cachexia.

Next, the supplementation of hydroxy methyl butyrate (HMB) also has been observed in cachexia models. HMB can modulate protein turnover because of its potential to induce protein synthesis through the activation of the mTOR pathway [4]. In a male Wistar rat model of cachexia with AH-130 ascites hepatoma inoculum, a diet with 4% HMB-enriched chow resulted in a 3% amelioration in cancer-induced muscle wasting and reduction in body weight [70]. The levels of phospho-mTOR and phospho-S6K were increased in HMB-treated groups, indicating improved muscle protein anabolism. In a comparative study on the anti-catabolic effects of leucine and HMB in both cachexia-induced mice and C2C12 myotubes, 50 µM HMB was more effective in attenuating protein degradation than 1 mM leucine [71]. Apart from animal models, the role of HMB has also been shown in cancer patients. Interestingly, in a double-blind, randomized controlled trial, advanced stage (IV) patients with CC were given a combination of 3 g/d of HMB, 14 g/d of L-arginine, and 14 g/d of L-glutamine. The patients in the treatment group showed an increase in body mass (0.95 +/− 0.66 kg) and fat-free mass (1.60 +/− 0.98 kg) [72]. Furthermore, another study has demonstrated that supplementation with HMB/Arg/Gln resulted in less muscle breakdown in patients with gastric cancer [73]. However, these positive effects of HMB were not observed in all studies. For example, the combination of HMB/Arg/Gln could not reverse the lean body mass wasting among patients with lung, breast, and prostate cancer [74]. This discrepancy suggests that the efficacy of this amino acid combination may vary depending on the type of cancer being treated. Therefore, more clinical trials are needed to evaluate the efficacy of HMB in human patients with cachexia.

Glutamine, a conditionally essential amino acid, plays a key role in cellular function. It serves as a primary substrate for protein synthesis, providing nitrogen for the synthesis of non-essential amino acids and serving as a precursor for nucleotide synthesis. Moreover, glutamine’s significance extends to its involvement in activating the mTOR pathway, thereby facilitating protein synthesis and cellular growth [4]. Furthermore, it plays a crucial role in protecting mitochondrial function by activating glutathione peroxidase 1 (GPx1) and inhibiting ROS [75]. In cancer patients, the dysregulated glutamine metabolism often leads to impaired protein synthesis, ultimately contributing to muscle wasting [75]. It has been reported that the supplementation of glutamine helps in reducing inflammation by enhancing type-1 conventional dendritic cells (cDC1s)-mediated CD8+ T cell immunity [76]. Also, it has been observed that glutamine supplementation could mitigate muscle protein wasting and support lymphocyte proliferation in patients with cancer. In this context, a study has shown that oral glutamine supplementations of 30 g/day for 4 weeks in patients with advanced esophageal cancer was associated with reduced muscle protein loss. Additionally, it improved the systemic lymphocyte function in these patients [77]. The same study also showed the importance of glutamine supplementation in AH109A hepatoma cells inoculated tumor-bearing rats. Glutamine-supplemented total parenteral nutrition not only augmented the muscle protein synthesis rate but also mitigated the protein breakdown rate, thereby reducing total body weight loss in these tumor-bearing rats [77]. In addition, glutamine substitution showed an improvement in the energy balance and preventing tumor growth in Walker-256 tumor-bearing rats [78]. These studies highlighted the beneficial effects of glutamine supplementation in the context of cachexia, suggesting its potential intervention to improve muscle metabolism and energy balance and to inhibit tumor growth.

Also, arginine is one of the conditionally essential amino acids and is available to the body through dietary intake and also by de novo synthesis from citrulline. Arginine primarily enhances protein synthesis by activating the mTOR pathway and inhibiting protein degradation through the stimulation of mTOR phosphorylation in skeletal muscle [79]. Research indicates that arginine supplementation could ameliorate CC by supporting protein synthesis, thereby potentially improving patient outcomes [80]. Furthermore, arginine supplementation has been linked to enhanced immune function by increasing the production of interleukin-2 (IL-2) and promoting T lymphocyte responses [4]. Also, it has been demonstrated that arginine supplementation may inhibit tumor growth and metastasis in breast cancer by enhancing Th1 immune responses [81]. Nitric oxide (NO) is a crucial signaling molecule that plays a role in mitochondrial respiration and oxidative damage. Arginine supplementation has been shown to reduce ROS production by inducing NO, ultimately impairing tumor growth [81]. Further research on elucidating the role of arginine in tumor progression is crucial for understanding its potential impact on CC.

Studies have elucidated the involvement of tryptophan, an essential/conditionally essential amino acid, in the context of cancer-related cachexia. Multiple investigations indicate that tryptophan plays a crucial role in protein synthesis and exhibits specific anti-inflammatory properties. Tryptophan has been identified as a contributor to the activation of the mTOR pathway via IGF-1. By activating mTORC1, tryptophan enables the activation of p70S6K (p70 ribosomal S6 kinase) and 4E-BP1 (eukaryotic translation initiation factor (4E)- binding protein 1) molecules, which are well-known potent regulators of protein translation [82]. On the other hand, the modulation of tryptophan metabolism via the kynurenine pathway has been widely studied in the presence of cytokines. In this context, various cytokines, such as TNF-α and IFNγ, induce the activation of enzymes like IDO (indoleamine-2,3-dioxygenase)/TDO (tryptophan-2,3-dioxygenase), facilitating the conversion of tryptophan to kynurenine. Subsequently, kynurenine aminotransferases (KATs) catalyze the conversion of kynurenine into kynurenic acid, known for its anti-inflammatory properties through the suppression of cytokines, such as TNF-α and IL-4 [83]. Interestingly, there are a few studies conducted on the role of tryptophan in cancer patients [84], as well as in various murine models associated with cancer-induced cachexia, such as melanoma B16F10 and KPC-based pancreatic adenocarcinoma [85]. Plasma tryptophan levels decreased in B16F10 tumor-bearing mice even during the early stages of tumorigenesis. In KPC-injected mice, tryptophan levels fluctuated but were also reduced, while in cachectic patients, they was significantly lower, which was associated with the increased expression of atrophy-related genes, mainly Atrogin 1. In this study, the authors demonstrated that treatment with 1-methyl-tryptophan, a tryptophan degradation inhibitor, restored plasma tryptophan levels, increased protein synthesis, and alleviated systemic inflammation [85]. Moreover, another study has demonstrated the involvement of IL-6 in tryptophan metabolism alterations via the kynurenine pathway, contributing to muscle degradation among patients with intra-abdominal sepsis. Notably, the administration of IL-6-neutralizing antibodies showed a reduction in muscle wasting in these patients [86]. These studies highlighted the crucial role of tryptophan in mitigating CC and emphasized the importance of further investigations in this direction.

Carnitine, derived from lysine and methionine, plays an important role in the esterification of fatty acids in the mitochondrial membrane. It facilitates the conversion of fatty acids into acetyl-CoA through beta-oxidation by interacting with the carnitine shuttling system. Carnitine palmitoyltransferase-1 (CPT1) releases CoA to form an acylcarnitine, which is transported into the mitochondrial matrix. CPT-2 dissociates the acyl group from carnitine, links back to CoA to import long-chain fatty acids for beta-oxidation, and ultimately provides cellular energy. Studies have demonstrated that carnitine plays a key role in scavenging ROS and mitigating oxidative stress, by inducing GPx1, thereby protecting mitochondrial function [87]. Moreover, it possesses anti-apoptotic and anti-inflammatory properties, through its ability to reduce levels of CRP (C-reactive protein), TNF-α, and IL-6 [88]. Furthermore, carnitine supplementation has been found to stimulate protein synthesis by increasing the expression of IGF-1. Also, it exerts a protective effect by reducing protein degradation in skeletal muscle through the downregulation of MuRF1 and the ubiquitin-mediated proteasomal complex (UPS) [89]. Supplementing with carnitine may increase lean mass in individuals with chronic diseases [90]. Studies have shown that the supplementation of carnitine improves the loss of muscle mass in patients with CC [91]. In a randomized, multicenter, and double-blinded trial, 72 patients with advanced pancreatic cancer and cachexia syndrome received 4 g of oral carnitine supplementation for 12 weeks. The results showed significant improvements in body mass index, body fat, and total muscle mass, with an increase of 3.4  ±  1.4% [92]. In another study with 12 patients having mixed tumor types of stage III and IV, the consumption of 6 g carnitine per day for over 4 weeks resulted in a lean mass augmentation of over 10% [93]. In addition to patients with CC, carnitine supplementation has also been studied in various experimental models of CC. Interestingly, in a C26 CC model, mice fed with 18 mg/kg/day carnitine for seven days resulted in a significant increase in the weight of their gastrocnemius muscle, blood glucose levels, and serum albumin levels. Additionally, it led to a decrease in their total cholesterol level [94]. Moreover, the supplementation of 1 g/kg body weight carnitine to a model with cachexia that was induced by the inoculation of rats with AH-130 Yoshida ascites hepatoma cells resulted in the downregulation of atrogin-1 and MuRF1, as well as reduced proteasome activity in the gastrocnemius muscle [95]. These studies suggest that the administration of carnitine may be a beneficial approach for the treatment of CC. However, more molecular studies are needed to investigate the exact working mechanism.

## 4. The Application of Exercise and Amino Acid Supplementation in Managing Cancer Cachexia

**Figure 1:**
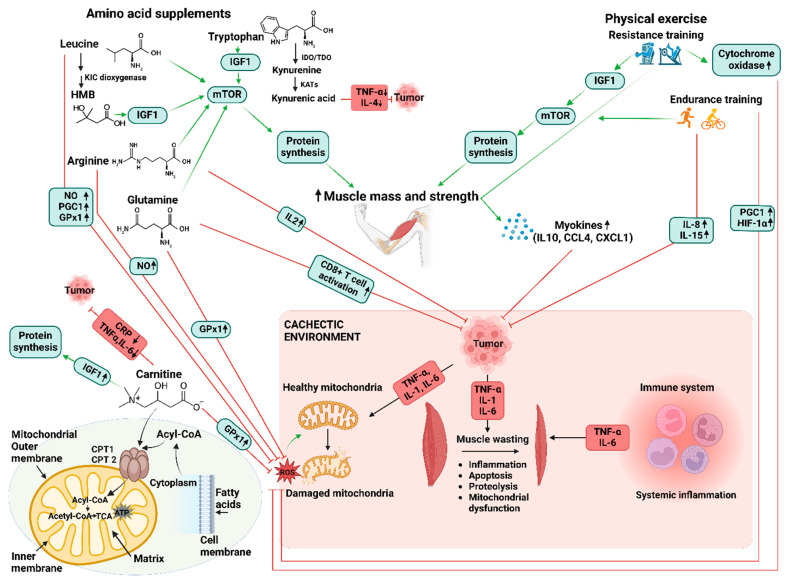
This schematic illustrates the intricate communication network involved in cancer cachexia. Cachexia-inducing tumors release various factors, including cytokines and other mediators, leading to inflammation and disturbances in mitochondrial metabolism. These disruptions directly trigger muscle atrophy. Furthermore, the figure highlights the potential of amino acid supplementation and physical exercise in ameliorating cancer cachexia-induced muscle wasting. Emerging research has shown the crucial role of specific compounds like carnitine. Carnitine plays a crucial function in energy metabolism by transporting fatty acids into mitochondria, where they are oxidized to produce energy, thereby counteracting the muscle-wasting process observed in CC. Stimulatory effects are depicted by green arrows, while inhibitory effects are represented by red lines. Upward arrows indicate upregulation and downward arrows indicate downregulation. Key factors and interventions are annotated: CCL4 (chemokine ligand 4), CD 8(Cluster of differentiation 8), CPT (carnitine palmitoyltransferase), CRP (C-reactive protein), CXCL1 (C-X-C ligand 1), ET (endurance training), GPx1 (glutathione peroxidase 1), HIF-1α (hypoxia-inducible factor 1α), HMB (β-hydroxy methyl butyrate), IDO (indoleamine-2,3-dioxygenase), IGF-1 (insulin-like growth factor 1), IL (interleukin), KATs (kynurenine aminotransferases), KIC dioxygenase (ketoisocaproate dioxygenase), NO (nitrogen oxidase), PGC1 (peroxisome proliferator-activated receptor–gamma coactivator 1), ROS (reactive oxygen species), RT (resistance training), TDO (tryptophan-2,3-dioxygenase), TNF-α (tumor necrosis factor-α), and WAT (white adipose tissue). This figure was created using BioRender.com.

In cancer cachexia, the combined effects of inflammation and oxidative stress alter both anabolic and catabolic signaling pathways, resulting in muscle atrophy, which ultimately affects the quality of life. Hence, the suppression of muscle atrophy is crucial for the quality of life and survival of patients with cancer. Regular physical exercise is linked to a lower risk of disease, as well as increased bone and muscle strength, leading to reduced mortality rates in healthy individuals. Moreover, moderate exercises are crucial in promoting metabolic and molecular adaptations, not only to enhance the health status of healthy individuals but also to benefit athletes [96]. Physical exercise combined with adequate nutrition is known to be beneficial for muscle hypertrophy. In particular, skeletal muscles adapt through exercises such as resistance training (RT) and endurance training (ET), which modulate de novo synthesis of myofibrillar proteins and mitochondrial proteins’ synthesis rates, respectively [97]. Moreover, exercise has been shown to affect not only the skeletal muscle but also various other organs [98,99]. Some studies have highlighted the beneficial outcomes from the combination of exercise and amino acids supplementation, in both healthy individuals and patients with CC. These insights are further described in the subsequent sections. The advantageous effects of amino acid supplementation and exercise in enhancing protein synthesis and reducing oxidative stress, inflammation, and immunosuppression are depicted in Figure 1.

## 5. Exercise and Amino Acids in Healthy Individuals

### 5.1. Resistance Training and Amino Acids

Skeletal mass is maintained when muscle protein synthesis (MPS) exceeds muscle protein breakdown (MPB), which leads to a positive net protein balance (NPB), i.e., the algebraic difference (MPS minus MPB). RT is considered the most effective stimulus in increasing skeletal muscle mass, functionality, and strength [100]. However, an intensive single session of RT causes the elevation of both MPS and MPB, resulting in a negative NPB [101]. Supplementing with proteins or amino acids after resistance training has a synergistic effect on muscle protein synthesis and results in a positive net protein balance [102]. Notably, the metabolic demand for protein intake is increased in athletes compared to non-exercising peers, which is why a 1.5- to 2.0-fold higher protein intake than the recommended daily intake of 0.8 g/kg/d is advised for athletes [103,104,105]. Moreover, the immune system is transiently suppressed immediately after intensive exercises, making it more prone to infection, which may last from 6 h to 1 week post-exercise, based on the exercise intensity [106]. This may be due to changes in amino acid metabolism after intense exercise, ultimately resulting in immunosuppression.

Exercise and amino acids supplementation induce interorgan crosstalk between mitochondria and skeletal muscles, leading to metabolic changes in skeletal muscles. In this context, it has been demonstrated that the expression of mitochondrial PGC1α is increased by exercise, which then activates BCAA metabolism, fatty acid oxidation, and the Krebs cycle and raises energy expenditure [107,108]. Physical exercise and BCAA administration together improved muscle mass and strength in older individuals, compared to leucine administration alone [109,110]. Several studies showed the impact of BCAAs on muscle anabolism when supplemented during and/or after exercise in healthy human subjects, e.g., they mediate signal transduction through S6K in skeletal muscle [111], activate mTOR kinase [112], and stimulate myofibrillar muscle protein synthesis [113]. Furthermore, the intake of BCAA-rich food and RT improved muscle mass and strength in older women with sarcopenia [109]. Also, leucine-rich AA supplementation for 12 weeks before and after RT reduced muscle loss in elderly participants [114]. Other amino acids are also frequently tested on individuals who were active in sports. Pre- and post-exercise EAA supplementation attenuated the loss of muscle mass and strength compared to AA intake alone [115,116]. Also, HMB has been demonstrated to have positive effects on muscle strength and fat-free mass when combined with RT in young adults [117]. Considering other amino acids, an experiment was conducted where a group of young males (aged 19 ± 1.58 years) performed RT after taking an amino acid cocktail comprising arginine, histidine, isoleucine, leucine, lysine, methionine, phenylalanine, valine, aspartate, glutamine, and tyrosine. The same cocktail was given to the same group again after the exercise. The group who took the amino acid cocktail showed increased muscle protein synthesis, anabolism, and performance compared to the group who received a carbohydrate placebo solution [118].

In addition to human studies, the beneficial effect of AA supplementation in combination with exercise has also been observed in animal studies. For instance, a study showed that rats who received six weeks of BCAA supplementation along with exercises showed an increase in glycogen levels in both their liver and muscles [119]. Furthermore, another study showed that supplementation of BCCA helps to maintain muscle fiber size and enhance physical endurance and motor coordination in middle-aged mice and also increases the expression of PGC-1α and sirtuin 1 (SIRT1), which promotes the growth and function of mitochondria in skeletal muscles through the regulation of mTORC1 [67]. Nevertheless, some studies concluded that BCAAs showed no effect on performance, heart rate, or fatigue prevention after RT [120,121,122,123]. Therefore, more studies should be undertaken on the ergogenic effects of BCAA supplementation. Stabilization of the muscle strength of mice was also shown with the combination of RT and HMB supplementation [124]. Furthermore, L-arginine supplementation and aerobic training in rats improved muscle anti-oxidant properties [125] and, along with RT, increased muscle mass and reduced DNA damage [126]. A combination of glutamine and alanine supplementation followed by resistance training resulted in less fatigue [127]. In summary, AA supplementation in combination with RT has been shown to improve muscle strength and reduce muscle damage.

### 5.2. Endurance Training and Amino Acids

Endurance training (ET) generally refers to aerobic training like walking, running, cycling, swimming, and bodyweight exercises including squats, push-ups, lunges, etc., which increase endurance. It has been proposed that AA supplementation coupled with ET increases lean body mass and muscle strength, improves fatigue and post-exercise recovery [128], and also modulates mitochondrial protein synthesis rate in both young and older adults [129,130]. Interestingly, ET has been linked to amino acid oxidation, specifically the oxidation of BCAAs. It is speculated that the intake of BCCA during low- or high-intensity endurance training can increase muscle protein synthesis and, thus, strengthen performance [128]. The performance of ET depends on factors like maximum oxygen consumption (VO_2max_) and the sustenance of the VO_2max_ percentage during ET. There is a difference in energy and metabolic activity based on short-term or high-intensity ET. Repeated sprint training is recognized as a potent form of multifaceted training, demonstrated to enhance VO_2max_. Notably, it has been demonstrated that combining high-intensity sprint interval training with both protein and carbohydrates can enhance protein synthesis [131]. Moreover, it has been noted that a combination of protein and carbohydrates with high-intensity ET leads to an improvement in overall protein balance [132]. However, a study has found that combining protein intake with high-intensity ET exercise did not result in an improvement in muscle damage [133]. Limited studies observed an impact of dietary protein ingestion after high-intensity ET on mitochondrial protein synthesis. For instance, PGC-1α mRNA expression and mitochondrial recovery were increased after ET with a 6 h post-exercise carbohydrate and whey protein co-ingestion for 2 weeks [134].

The average amount of EAA supplementation in combination with aerobic ET leads to enhanced muscle strength, functionality, and protein metabolism [135,136]. Other studies have shown that acute and chronic BCAA supplementation in endurance athletes modified exercise-mediated immune suppression [137] and suppressed muscle damage or delayed-onset muscle soreness [138]. During ET, BCAAs had protein-sustaining effects on human subjects either by promoting protein synthesis or suppressing protein degradation [139]. It could be shown that HMB supplementation can improve endurance performance and VO_2max_. It has the potential to increase the gene expression of PGC-1α, which in turn can increase mitochondrial biogenesis and oxidative metabolism capacity [140]. Next, there are some studies have shown the importance of glutamine supplementation in improving muscle damage during periods of high-intensity ET [141]. Also, there is some evidence suggesting that glutamine might play a role in promoting anabolic processes, such as muscle glycogen and protein synthesis, contributing to enhanced muscle recovery [142,143]. Altogether, the above studies showed that the combination of AAs and ET can synergistically restore muscle mass in adults.

### 5.3. Exercise in Managing Cancer Cachexia

To study the effects of exercise on protecting CC-mediated skeletal muscle deterioration, impairment, oxidation, and inflammation, several in vitro and in vivo techniques have been developed in the past years, including electrical pulse stimulation (EPS) in cell culture models and cachectic laboratory animal (mice, rats) models, respectively [144,145]. EPS is an in vitro model where muscle contraction is mimicked in cultured skeletal muscle cells of human and animal origin. It has been used to investigate the role of skeletal muscles and their crosstalk with other tissues, the regulation of metabolic functions, signaling events, tissue engineering, and so on [146,147]. EPS is a tool to detect novel myokines, and cytokines released from muscle cells, and to identify the connection between exercise and well-being [147,148]. Two types of EPS, including acute high-frequency and chronic low-frequency, are generally used in myotubes. In acute high-frequency EPS, single bipolar pulses of 2 ms, 100 Hz for 200 ms every fifth second for 5–60 min, and 10–30 V are applied on differentiated myotubes [149,150]. This produces similar conditions to acute high-intensity exercises and results in increased cellular glucose uptake, increased lactate production, and decreased cellular ATP content [146,151]. On the other hand, chronic low-frequency EPS uses single bipolar pulses of 2 ms, 1 Hz, and 30 V continuously for the last 24–48 h during a myotube differentiation period [151]. This low-frequency EPS technique has been proven to increase glucose uptake, mitochondrial content, cellular oxidation, citrate synthase activity, insulin sensitivity, activation of AMPK, IL-6, and anti-inflammatory activity [146,151,152]. Different EPS protocols exist today, and new ones are continuously being developed to mimic various forms of exercise training, and each has its pros and cons [153]. Although EPS cannot replicate all the conditions of in vivo exercises, as it lacks motor neurons, neuromuscular junctions, blood flow, and innervation, it still has great potential in the study of muscle contraction in myotubes in vitro. Moreover, further advancements in the technique could improve our understanding of the positive effects of exercise.

Various studies were performed on rat or mouse models of CC to explore the effects of RT and ET on skeletal muscle metabolism. Various controversial studies exist regarding the benefits of moderate and intense treadmill training in CC. For instance, a study has demonstrated when moderate and intense treadmill training were compared in a cachectic mice model, only the intense treadmill training improved the quality of life, survival rate, and controlled muscle atrophy [154]. In contrast, another study has shown that 8 weeks of moderate treadmill exercise prevented IL6-induced cachexia in APC^min/+^ mice and improved insulin sensitivity, muscle oxidative capacity, and muscle metabolism [155]. Interestingly, low-intensity ET also restores loss of muscle mass by suppressing the ubiquitin–proteasome pathway, increasing hypoxia-inducible factor-1α (HIF-1α), phospho-AMPK, and activating the mTOR pathway in soleus muscle, which prevented CC-induced muscle atrophy in male Wistar rats [156]. Additionally, voluntary exercise in mice with C26-induced colon carcinoma has been found to trigger autophagic degradation, which helps to restore muscle function and prevent cancer cachexia [157]. Furthermore, voluntary wheel running in C26-tumor-bearing mice counteracted Pax7 overexpression and NF-κB activation, thereby rescuing muscle mass and fiber size [158] and also effectively countered mitochondrial dysfunction to mitigate muscle loss in cachexia [159]. Surprisingly, voluntary wheel running did not improve body weight, muscular properties, or functional capacity in a C26-induced cancer cachexia mouse model, nor did it inhibit tumor growth [160]. Recently, it has been demonstrated that aerobic exercise successfully attenuated muscle atrophy, activated adiponectin signaling, increased mTOR phosphorylation, and suppressed light chain 3 (LC3-II) in a C26-induced cachexia model and C2C12 myotube model of cancer cachexia [161]. Some studies have also demonstrated that combined RT and ET can improve muscle mass and function in a C26-induced cachexia model. For example, one study has demonstrated that combined RT and ET can increase muscle mass and strength while regulating autophagy and proteasome-mediated degradation [162]. Another study has shown that combined RT and ET can mitigate the reduction in muscle mass in C26-induced cancer cachexia by reducing systemic inflammation [163]. These pre-clinical studies on animal models suggest potential benefits to combat CC, including increased protein synthesis and improved muscle wasting from both RT and ET.

Due to their antioxidant and anti-inflammatory functionalities, both RT and ET have the potential to be used as therapeutics in patients with cachexia [164,165,166]. Several studies have described the beneficial impact of exercise training during [167,168,169,170,171,172,173] and after [174,175,176,177] cancer treatment on attenuating fatigue, boosting performance, and enhancing the quality of life. Among them, ET has been shown remarkable benefits, including mitigating cancer-related fatigue [178], increasing anti-inflammatory markers such as IL-8 and IL-15 [179], enhancing muscle strength, promoting antioxidant capacity, and decreasing oxidative stress markers [180]. Meanwhile, RT has been shown to prevent cancer-induced myofiber atrophy, reduce inflammation by reducing the expression of TNF-α and IL-6 [181], and mitigate oxidative damage by inducing cytochrome oxidase [182]. The functional effect of RT and ET normally can vary from cancer to cancer. For instance, in our study, we have shown that RT in pancreatic cancer patients stimulates the release of myokines CXCL1, IL10, and CCL4 from skeletal muscle. These myokines not only inhibited the growth and migration of pancreatic tumor cells but also induced cell death of tumor cells in vitro [183]. Moreover, a randomized controlled trial conducted on patients with pancreatic cancer demonstrated that RT enhances body weight by 3.2% and muscle strength by 30% [184]. Similarly, a review focusing on patients with pancreatic cancer demonstrated that ET can also significantly improve muscle strength [185]. In addition, in men receiving radiation therapy for prostate cancer, both RT and ET contributed to the reduction in fatigue by improving muscle strength and body mass [186]. Furthermore, in a study of breast cancer patients who received adjuvant chemotherapy, it was found that neither RT nor ET had a significant effect on muscular strength and lean body mass, but both training methods improved their physical fitness, self-esteem, and body composition [187]. Similarly, patients with head and neck cancer who underwent RT experienced a noticeable improvement in general fatigue and quality of life [188]. Also, a randomized controlled trial discovered that both RT on its own and in combination with ET caused a significant increase in peak oxygen uptake (VO_2_ peak) for lung cancer patients, leading to improved muscle strength [177]. A deep understanding of the key roles played by RT and ET in protein synthesis and muscle mass can offer significant benefits for patients with CC.

## 6. Synergistic Effects of Combined Application in Managing Cancer Cachexia

Although several studies suggested the intake of nutritional supplements, particularly in the form of proteins or AAs [189,190,191,192], some reported that nutritional interventions alone are not sufficient to counteract CC or reduce cancer-mediated mortality [190,193,194,195]. In addition to AA supplements, exercise intervention has been suggested as an efficient treatment strategy to antagonize CC and attenuate fatigue in advanced cancer patients [189,196,197,198,199,200]. In this context, research has elucidated the efficacy of combining RT with EAA supplementation in restoring muscle protein metabolism in sarcopenia [201,202]. The incorporation of BCAAs, particularly leucine, with resistance training has demonstrated positive effects on reducing muscle wasting induced by various types of cancer [203]. Regarding other AAs, it has been shown that the supplementation of arginine along with RT resulted in significant protein synthesis and attenuating muscle wasting in patients with cancer [204]. The literature on ET combined with AA supplementation is relatively limited; it has been noted that this approach could contribute to improvements in weight loss, fatigue, and quality of life [13,205]. Summaries of preclinical studies involving rats or mice and humans, investigating the effects of RT and ET in combination with AAs on skeletal muscle functionality and associated signaling pathways in cancer and cachexia models, are provided based on evidence from both animal and human studies (Table 1 and Table 2, respectively).

Individuals with advanced cancer often face significant physical weakness and are frequently burdened by the adverse effects of cancer therapies, such as chemotherapy and radiation. These factors can severely limit their ability to participate in strenuous exercise training. To address this issue, we utilized a combination of two innovative training methods, whole-body electromyostimulation (WB-EMS) and protein-rich nutritional therapy, in advanced cancer patients receiving oncological treatment [12,211]. Our findings revealed that using WB-EMS alongside a high-protein diet significantly improved muscle wasting by increasing skeletal muscle mass, body weight, physical function, and performance status in these patients [12]. Given its effectiveness, WB-EMS can be considered a potential physical exercise therapeutic option for individuals with advanced cancer.

Recently, there has been a growing consensus suggesting multimodal interventions by integrating exercise, nutritional therapy, and pharmacological interventions, consisting of non-steroidal anti-inflammatory drugs (NSAID) and eicosapentaenoic acid (EPA), to synergistically target cachexia [212,213]. Only a restricted number of studies have analyzed this approach. A retrospective study conducted on 374 patients with CC showed that those who had experienced weight loss within the last 6 months and an impaired quality of life benefited significantly from a multimodal approach [214]. However, further research on multimodal approaches, especially the implementation of amino acids in strength-based training is needed to improve the quality of life of CC patients.

## 7. Future Perspectives

To counteract CC, improvements in muscle mass, strength, functionality, and muscle protein synthesis are crucial. Many in vitro and in vivo studies evaluated the effects of amino acids on increasing muscle protein synthesis and decreasing protein degradation in cachectic patients. However, clinical trials or pilot studies on cachectic patients still need more information on the appropriate timing, dosage, therapy duration, and most importantly, which amino acid combination plays a vital role. A common occurrence is patients often drop out before the trial ends. Also, each cancer type behaves differently, and therefore, the application of therapeutics among different cancer types and cachexia stages should be taken into consideration during the planning of experiments or clinical studies.

Numerous studies have shown encouraging outcomes of exercise in individuals with advanced cancer. These findings suggest that exercise could potentially play a key role in patients with advanced stages of cancer. However, it is still not specified which exercise types, intensity, frequency, and duration are most appropriate for individualized patients. The exercise showed more impact when started in the pre-cachexia stage compared to the cachexia stage and was not feasible in the refractory stage. Therefore, to prevent cachexia, more complementary treatment strategies should be developed concerning exercise training. On the other hand, exercise combined with various amino acids has shown some success in experimental cancer cachexia treatments. However, there are a limited number of studies, and more research is needed to determine the optimal combination. In future studies, it is important to pay attention to the interaction between different components of the multimodal approach. The combination should contribute to therapy effectiveness while also ensuring the safety of cachectic patients.

## 8. Conclusions

In summary, CC multifactorial syndrome is characterized by the loss of skeletal muscle mass and fat tissue. The intricate interplay of inflammation, oxidative stress, and metabolic dysfunction further exacerbates the progression of cachexia. While therapies are being actively investigated in both preclinical and clinical settings to address these fundamental mechanisms, their efficacy remains uncertain. Nonetheless, strategies that include amino acid supplementation and physical exercise have shown mitigation of muscle loss and improvement in overall patient outcomes. Notably, certain studies suggest multimodal approaches, wherein combining dietary supplements, exercise, and pharmacotherapy is a more comprehensive approach to managing cachexia, although determining the optimal combination remains unclear. Further research in this direction is needed to elucidate optimal treatment protocols and improve therapeutic strategies for combating CC effectively. By targeting the molecular pathogenesis of cancer-associated cachexia and utilizing the synergistic effects of exercise and amino acids, we can facilitate the development of more effective interventions and improve the quality of life for patients with CC.

## Figures and Tables

**Table 1:** Studies on nutrition and/or exercise in animal models.

Experimental Models and Sex	Exercise Types	Treatment (Dosage, Duration, Route)	Result	Reference
(M) Wistar rats	Moderate intense training @ 5 d/wks for 8 wks	300 mg/kg L-Arg in 30 mL DW daily for 8 wks	↓ plasma cholesterol, VEGF, CINC, triglycerides	[206]
(M) Wistar rats	Swimming for 6 wks @ 60 min/day	3.57% and 4.76% BCAAs in two groups for 6 wks	↑ hepatic and muscle glycogen	[119]
(M) Mice	Treadmill running @ 5 days/wk for 4 wks (1st wk, 30 min at 10 m/min; 2nd wk, 60 min at 10 m/min; 3rd and 4th wk, 60 min at 12 m/min)	BCAAs @ 1.5 mg/g body weight/d in drinking water	↑ SOD, CAT, GSH-PX	[67]
(M) LLC mice model	HIIT; each session: five intervals of 3 min of treadmill running @ 18 m/min, then 4 min of running @ 25 m/min; 16 days	No additional treatment	↓ tumor progression, ↑ survival rate, running capacity, skeletal muscle contractility	[207]
(M) C-26 mice model	Moderate exercise: 0.5 km/h, 70% maxHR; severe exercise: 1 km/h, 90% maxHR; Both 45 min/d, once every two days for 4 wks	No additional treatment	↓ muscle atrophy, ↑ QoL, survival rate	[154]
(F/M) Apc^Min/+^ mice model	Moderate exercise: 18 m/min, 1 h, 6 days/wk	No additional treatment	↓ IL-6-dependent cachexia status ↑ insulin sensitivity, muscle metabolism, oxidative capacity	[155]
(M) AH130-induced rat model	Low-intense exercise: 15 m/min, 30 min/session, 1 wk	No additional treatment	↓ ubiquitin-proteasome pathway, cachexia-induced muscle atrophy ↑ HIF-1α, phospho-AMPK, mTOR pathway	[156]
(F/M) TRAMP mice model	Voluntary wheel running, 20 wks	No additional treatment	↓ myostatin level ↑ muscle mass, forelimb grip force	[208]
(F) C-26 mice model	Voluntary wheel running, 19 days	No additional treatment	↓ atrogene induction, autophagic flux, cachexia ↑ muscle mass, muscle homeostasis	[157]
(F) C-26 mice model	Voluntary wheel running, 19 days	No additional treatment	↓ Pax7 overexpression, NF-κB activation, cachexia ↑ muscle mass, fiber size	[158]
(M) C-26 mice model	Combined training; RT: climbing 1 m ladder inclined at 85°, ET: wheel running, 25 min, 5–9 m/min for 5 days	No additional treatment	↓ autophagy (LC3B-I/II ratio), cachexia ↑ muscle mass, strength	[162]
(M) Walker-256 rat model	RT, voluntary ladder climbing, 12 days	No additional treatment	↓ muscle wasting, oxidative stress, inflammation	[209]

DW: drinking water; VEGF: vascular endothelial growth factor; CINC: cytokine-induced neutrophil chemoattractant; LLC: Lewis lung carcinoma; C-26: Colon-26; HIIT: high-intensity interval training; maxHR: maximum heart rate; QoL: quality of life; Apc: adenomatous polyposis coli; AH130: AH130 Yoshida ascites hepatoma cells; HIF-1α: hypoxia-inducible factor-1α; AMPK: AMP-activated protein kinase; mTOR: mammalian target of rapamycin; TRAMP: transgenic adenocarcinoma of the mouse prostate; NF-κB: nuclear factor kappa B; RT: resistant training; ET: endurance training; F: female; M: male; F/M: female/male; wks: weeks. The downward arrow indicates downregulation and the upward arrow indicates upregulation.

**Table 2:** Studies on nutrition and exercise in humans.

Participants and Sex	Exercise Types and Duration	Study Types	Result	Reference
42 [135] or 50 [136] healthy adults (F/M)	For 22/24 weeks; 15 g EAA or placebo daily, ET (progressive vigorous treadmill walking 3 times/wk)	Randomized controlled trial	ET improved insulin sensitivity [135]; ↑ muscle protein synthesis [136]	[135,136]
12 healthy adults (F)	BCAAs (Ile:Leu:Val = 1:2.3:1.2), seven sets of 20 squats/set with 3 min intervals between sets	Randomized controlled trial	↑ serum myoglobulin by exercise but not BCAA; BCAA suppressed muscle damage	[138]
7 healthy adults (M)	Ergometer cycle exercise, 60 min on cycle ergometer, semirecumbent position, work rate 164 ± 7 W, ~75% VO_2max_	Non-randomized controlled trial	After exercise, the protein-sparing effect	[139]
65 pancreatic cancer patients (F/M)	Supervised progressive RT (RT1), home-based RT (RT2), and control; two times RT/wk, 6 months	Randomized controlled trial	RT1 improved elbow flexor/extensor and knee extensor muscle strength	[184]
121 prostate cancer patients (F/M)	RT or ET, RT: two sets of 8–12 repetitions of 10 different exercises, three times/wk for 24 wks; ET: 50–60% VO_2_ peak for 1–4 wks, then 70–75% for 5–24 wks	Randomized controlled trial	Both RT and ET reduced fatigue; RT ameliorated muscle strength, triglycerides, and body fat	[186]
242 breast cancer patients (F/M)	RT or ET, adjuvant chemotherapy to usual care (n = 82), supervised RT (n = 82) or supervised ET (n = 78); 17 wks	Randomized controlled trial	No improvement in QoL; both RT and ET improved self-esteem, physical fitness, and body composition	[187]
20 head–neck cancer patients (F/M)	Progressive RT (n = 10), usual care (n = 10); 3 × 30 min/week; 7–8 wks post-radiotherapy	Randomized controlled trial	RT improved fatigue and QoL	[188]
131 advanced cancer patients (F/M)	Usual care control with individualized nutrition (n = 35), intervention (n = 96); RT (20 min WB-EMS session, bipolar, 85 Hz, 2×/wk, 12 wks)	Non-randomized controlled trial	↑ skeletal muscle mass, body weight, physical function, and performance status by RT	[12]
9 colorectal cancer patients (F/M)	RT or ET, patients were given protein-rich meals. RT: consisted of 20 repetitions (60–65% 1-RM) followed by two sets of six repetitions (80–85% 1-RM); ET: consisted of 30 s and 60 s intervals with a 1:3 work–recovery ratio. Sessions were 60–75 min long, 3 times/wk for 4 wks.	Non-randomized controlled trial	Patients demonstrated a compliance rate of ≥80% with the exercise training program and nutritional intervention	[210]

ET: endurance training; RT: resistant training; QoL: quality of life; WB-EMS: whole-body electromyostimulation; RM: repetition maximum; F: female; M: male; F/M: female/male; wks: weeks. The upward arrow indicates upregulation.
